# Research on mechanical properties of weak expansive soil under conditions of different confining pressures and moisture contents based on triaxial test

**DOI:** 10.1371/journal.pone.0308364

**Published:** 2024-09-10

**Authors:** Xuelei Cheng, Qiqi Li, Ran Hai, Shunqun Li, Xianfeng He

**Affiliations:** 1 School of Architectural and Civil Engineering, Zhongyuan University of Technology, Zhengzhou, Henan, China; 2 Department of Civil Engineering Mechanics, Yellow River Institute of Hydraulic Research, Zhengzhou, Henan, China; 3 School of Civil Engineering, Tianjin Chengjian University, Tianjin, China; University of Sharjah, UNITED ARAB EMIRATES

## Abstract

Objective: To explore the mechanical properties of weak expansive soil under different moisture contents and confining pressures. Methods: The triaxial remolded soil samples with different moisture contents (14%, 16%, 18%, and 20%) were prepared for the obtained Nanyang weak expansive soil. Four groups of 16 consolidated drained triaxial shear tests with a confining pressure of 50, 100, 200, and 300 kPa were carried out to study the effects of confining pressure and water content on the deviatoric stress and pore pressure of weak expansive soil with strain. Results: The study showed that the shear strength of weak expansive soil samples gradually increased with the increase of the water content, but its increase nonlinearly decreased as the confining pressure increased. When the axial strain was small, the pore pressure significantly increased, and the pore pressure increased tended to be stable slowly and gradually as the axial strain gradually increased. Therefore, the research results could be used to avoid the diseases caused by the characteristics of weak expansive soil in engineering construction and provide theoretical references for improving engineering foundations in weak expansive soil areas.

## 1 Introduction

Expansive soil is widely distributed in China. As a special soil, expansive soil is a water-sensitive material, with the characteristics such as water absorption expansion and water loss shrinkage [1]. In recent years, the problem of waterlogging caused by precipitation has become more and more serious, and the damage to expansive soil foundations caused by the increase in soil moisture content has become more and more severe [2]. The infiltration of surface water into the deep soil causes the expansion of the foundation soil, resulting in slope slip, roadbed cracks, collapse, instability, etc. [3]. As for the foundations of expansive soil with serious water expansion, they are usually improved by physical, chemical, and biological methods to inhibit the expansion effect [4]. However, there are weak expansive soils in Nanyang and other areas of Henan Province. Whether it is necessary to improve the weak expansive soil foundations or which improvement method should be adopted needs to be studied from the perspective of the mechanical properties of weak expansive soil. Therefore, to solve pavement engineering problems, it is significant to study the mechanical properties and deformation characteristics of weak expansive soil [5].

As for the studies on the mechanical properties of expansive soil, Wang Huan [6] and Zhuang Xinshan [7] analyzed the mechanical properties of improved soil samples based on triaxial tests, and they found that the shear characteristics and cementation ability of the improved expansive soil had been enhanced to some extent. Li [8] discussed the meaning and nature of the strain state from a theoretical perspective, revealed the yield process of the material, and established the constitutive model, which provided a theoretical basis for mechanically analyzing the deformation of weak expansive soil. Niu Geng [9, 10], taking weak expansive soil as the test material, carried out the experimental study on the water retention and strength characteristics of unsaturated soil in a wide range of suctions, and then proposed a strength model of unsaturated soils suitable for a wide range of suction. Bao Yiyong [11] and Cheng Xuelei [12] analyzed the influence of rainfall on weak expansive soil foundations by using the soil-water characteristic curve test and expansive soil foundation reconstruction technology, providing a research basis for the study on the mechanical properties of weak expansive soil under different working conditions. Koteswaraarao [13] and Zakarka [14] employed triaxial tests to acquire the relationship between the shear strength parameters and the generalized plastic shear strain of expansive soil and then studied the impact of multiple factors on the compression index of expansive soil to analyze the slope stability of expansive soil. Yang [15] conducted experimental research on the liquid-plastic limit, particle distribution, and free expansion rate of Nanyang’s weak expansive soil, providing a basis for studying triaxial tests of weak expansive soil. Wu [16, 17] studied the results of a series of *K*_0_ consolidated undrained triaxial tests on soils under different stress histories and strain rates. Zheng [18, 19] and Gao [20] used a triaxial apparatus to study the strength and deformation characteristics of soil and the stress-strain relationship and volume change of expansive soil in a wide range of suction. Meanwhile, Zhang Zhiyuan [21] and Chen Xian [22] used finite element software to simulate the stress and strain of large-scale direct shear test results and the expansive soil foundation. Moreover, Víctor [23] and Bouatia [24] proposed a formula for a double structure model and simulated the hydraulic characteristics of expansive soil structure. According to the conversion relationship between expansive soil saturation and temperature and thermal expansion coefficient, Xiao Jie [25] used FISH software to simulate the hygroscopic expansion effect of soil in real time, which realized the three-field coupling of seepage field-displacement field-stress field of expansive soil slope when considering the three factors (groundwater level, permeability coefficient, and strength attenuation). Finally, Jiang Jie [26] carried out the model test of single piles under horizontal load of the condition of expansive soil before and after soaking and then analyzed the change laws of expansive soil foundations by using finite element software. The above research results revealed that there were diversified research methods for the mechanical properties of expansive soil, and different research methods should be adopted for different working conditions.

In summary, many scholars have studied the mechanical properties, deformation characteristics, and improvement measures of expansive soils in various regions based on triaxial tests and numerical simulations. As the water invades the soil from different directions in actual projects, the amount of groundwater infiltration will change the water content of the soil, and the influence of groundwater infiltration on the soil at different depths is not the same [27]. There are usually engineering problems in expansive soil areas under rainfall conditions [28, 29]. Researchers have analyzed the mechanisms revealed by experimental data from the perspectives of particle mechanics or fracture mechanics, providing ideas for the study of weakly expansive soils [30–33]. Meanwhile, there are relatively few studies on weak expansive soil from the perspective of different confining pressures and moisture contents, and there hasn’t been a clear enough influence mechanism on the shear strength and excess pore water pressure of weak expansive soil under different confining pressures and moisture contents, so it is necessary to strengthen the research on the mechanical properties of weak expansive soil. Therefore, this paper exployed the triaxial test to study the mechanical properties of weak expansive soil under different confining pressures and moisture contents.

## 2 Indoor test method

### 2.1 Test instrument

**Fig 1:**
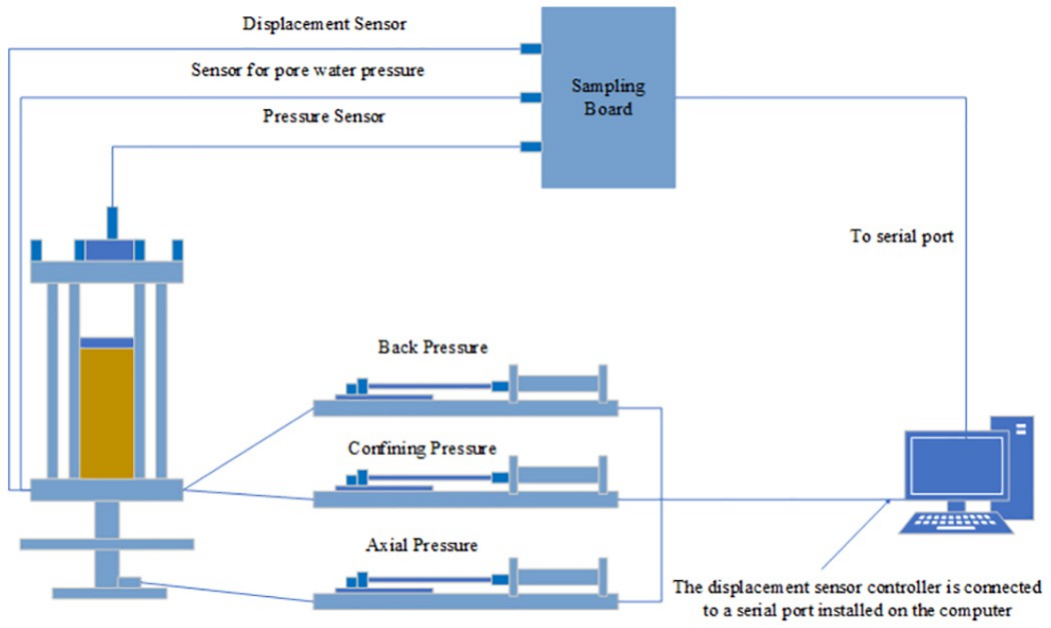
GDS triaxial apparatus.

This experiment was completed at the Geotechnical Laboratory of Zhongyuan University of Technology. The standard stress path triaxial test system (Fig 1) produced by the British GDS Instruments and Equipment Co. Ltd. was used in this test. The equipment mainly comprises a pressure chamber, a pressure system, and a measurement and acquisition system. Four sets of pressurization systems provide confining pressure, axial force, back pressure (water), and back pressure (gas), respectively. The measurement and acquisition system includes various sensors, such as built-in underwater load sensors and linear displacement sensors. The data acquisition board and converter were used for data acquisition and experimental control of GDSLAB module software. All measurement data were collected by the acquisition system. The measurement accuracy of the experimental equipment is as follows. Axial load: 8kN; Confining pressure: 2MPa; Sample size: 38×76mm/50×100mm; Pressure accuracy: <0.15%; Pressure resolution: 1kPa; Volume accuracy: <0.25%; Volume resolution: 1mm^3^; Axial displacement: ±25mm; Pore water pressure: 2MPa.

### 2.2 Specimen preparation

The samples were prepared based on *the Standard for Soil Test Methods* (GB/T50123-2019) [34]. Firstly, the loose weak expansive soil sample was dried and ground, and then under the same dry density, the soil sample was sieved using a sieve with a pore size of 2mm to configure the soil with the same mass as the soil sample. Secondly, the sieved dried soil was collected and placed in a plastic pot, and the water was sprayed with a spray bottle and stirred evenly. Finally, the mixed sample was put into a sealed bag and left to stand for 24 hours.

The produced soil samples were used for the compaction test, and an oil hydraulic jack was the compaction instrument. The soil samples were separated into three layers for compaction during the sample preparation process. The compaction height and the mass of each layer of soil sample were kept constant. Each soil layer was compacted, the surface was leveled, and the subsequent dirt loading and compaction were done to ensure that the soil particles were in good contact with one another. An extra 2 to 3 grams of soil samples were added to each layer prior to compaction because there was about 2 to 3 grams of soil lost during the sample preparation process. After the sample was made, its mass was weighted, and its actual dry density was calculated to ensure that the error between the sample and the actual set dry density was less than 0.01 g/cm^3^. Later, the sample was wrapped in a plastic wrap, marked, and placed in a moisturizing cylinder for use. *The Standard for Soil Test Methods* (GB/T50123-2019) stipulates that the sample is cylindrical, the minimum diameter is 33 mm, the average height-to-diameter ratio is between 2 and 2.5, and the maximum particle size should be less than 1/6 of the sample diameter. The GDS computer automatic triaxial device was used in the test, with a diameter of 50 mm and a height-to-diameter ratio of 2. Therefore, the sample size of this test was determined as follows: the diameter: 50 mm, the height: 100 mm. During sample preparation, divide the soil sample into three parts of the same quality and compact them in three layers. The thickness of each layer of the sample is 33.3mm. After each layer is compacted, record the height of the soil sample with a ruler to ensure that the compaction height is the same.

### 2.3 Test process

#### 2.3.1 Preparation stage

The residual air inside the pore pressure sensor was eliminated before the test. The pore pressure sensor, back pressure, and base were connected, so the air inside the pore pressure sensor was excluded by the back pressure controller. Specifically, the valve outside the base was opened, and the valve was closed when the base was out of water. At this time, the residual air inside the pore pressure sensor was discharged.

#### 2.3.2 Sample installation

First of all, to keep the two ends consistent, the rubber film was first placed inside the sampler. One end was tightened and aligned. After the other end was tightened and straightened without any internal folds, a pump balloon was used to pump air into the space. When loading, the base was wiped dry, the permeable stone and filter paper were put on the base, and then the soil sample was placed on the base. Then, the bottom of the rubber film of the soil sample was tightened, the rubber band was sheathed on the sampler, and the lower part of the rubber film was pulled to prevent the internal leakage of the soil sample. When installing the top hat, it was necessary to put on two rubber bands first, and then put on the filter paper. The top hat comes with permeable stone, and there is no need to install permeable stone. The soil sample shouldn’t be disturbed when loading the sample. When installing the belt, a three-lobe mold was used for protection, and then a stretching cap was installed. Finally, the pressure chamber was installed, the bottom of the pressure chamber was coated with alum to stop water, and then the pressure chamber was injected with water.

#### 2.3.3 Installation of pressure chamber

The exhaust valve at the top was opened, and then the inlet valve connected to the pump at the bottom was opened. Water was injected into the pressure chamber once the pump’s inlet valve was connected, and the inflow valve was shut once the pressure chamber was filled with water. Later, the pump plug was removed, and then the exhaust valve at the top was closed. The fixed valve at the top was spun after the top nut was turned counterclockwise until the top displacement sensor’s dowel bar made contact with the soil sample. At this point, the bottom pressure chamber’s inlet valve ought to be closed, the top exhaust valve should be closed, and the back pressure valve should be open.

#### 2.3.4 Operating steps

A soil sample with a diameter of 50 mm and a height of 100 mm was used, and data were recorded every 20 seconds. The data, including axial displacement, volume change, confining pressure, and so forth, should be reset before starting the test. The top cap within the pressure chamber was made to contact indefinitely by rotating the top nut of the chamber after the data was reset. A positive back pressure was first set before the confining pressure and axial pressure were adjusted for the test to prevent back pressure from absorbing water. The confining pressure used a linear loading method, and the axial pressure selected the axial compressive force. The axial pressure value was chosen to be 5 kPa higher than the confining pressure to guarantee that the top cap and the tensile cap made contact. When the test time reached the set value, the pressure was maintained and the saturation stage ended. After that, the confining pressure was linearly discharged by 0.4 kPa per minute while the consolidation stage test scheme was established and the axial and reverse pressures were maintained constant. The test duration was computed based on the specified confining pressure value. Finally, the test scheme of the shear stage was set, and the test ended when the axial deformation reached 15% of the height of the soil sample.

#### 2.3.5 End the test

After the triaxial test ended, the base should be lowered to remove the soil sample’s remaining axial pressure and confining pressure. Then the exhaust valve on the pressure chamber’s upper part was opened. Finally, the drainage valve on the base was opened. At this time, the liquid level inside the pressure chamber declined, and then the soil sample was taken out.

## 3 Test design scheme

The test soil was taken from Tanghe County, Nanyang City, Henan Province, and the physical properties of the soil sample are shown in Table 1. This test carried out a triaxial shear test on weak expansive soil under undrained conditions. The effects of different confining pressures (50, 100, 200, and 300 kPa) and different moisture contents (14%, 16%, 18%, and 20%) on the static shear characteristics of expansive soil samples were set up. The specific test scheme is shown in Tables 2 and 3.

**Table 1:** Basic physical and mechanical parameters of expansive soil.

Dry density/%	Free swell ratio/%	Natural moisture content/%	Maximum dry density/g·cm^-3^	Proportion	Liquid limit/%	Plastic limit/%
1.38	52	23.21	1.67	2.68	50.4	25.7

**Table 2:** Triaxial test scheme of expansive soil.

Test object	Dry density/%	Consolidation mode	Undrained test	Shearing rate mm·min^−1^	Moisture content (%)
Remoulded soil	1.38	Isotropic consolidation	CU50, CU100, CU200, CU300	0.4	14%, 16%, 18%, 20%

**Table 3:** Test design list.

Sample no.	Confining pressure (kPa)	Moisture content (%)
1	50	14
2		16
3		18
4		20
5	100	14
6		16
7		18
8		20
9	200	14
10		16
11		18
12		20
13	300	14
14		16
15		18
16		20

Note: CU50, CU100, CU200, and CU300 in the table represent the undrained tests of remolded soil with a confining pressure of 50 kPa, 100 kPa, 200 kPa, and 300 kPa, respectively.

## 4 Analysis of test results

### 4.1 Deviatoric stress-strain relationship

**Fig 2:**
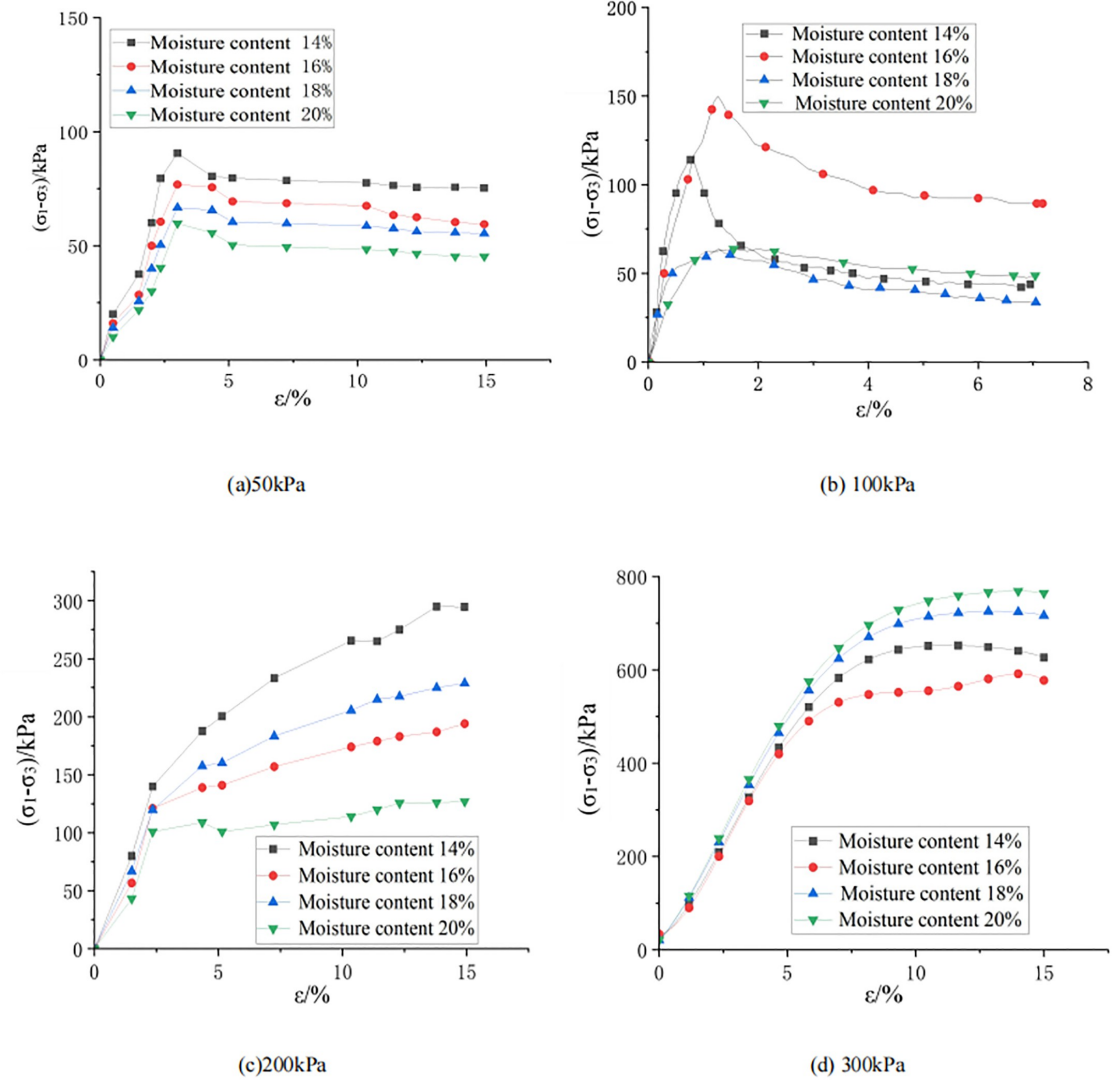
Stress-strain diagrams under different confining pressures.

Fig 2 shows the relationship curve between the axial strain and deviatoric stress of weak expansive soil samples under different confining pressures and different moisture contents, and it can be seen that the curves show roughly the same trend under different confining pressures and moisture contents, that is, as the axial strain increases, the deviatoric stress increases first and then decreases. As shown in Fig 2(a), when the confining pressure is 50 kPa, as the axial strain increases, the shear strength of the weak expansive soil sample increases first and the deviatoric stress reaches a peak at an axial strain of about 3%, then decreases and tends to be stable. As the moisture content increases, the deviatoric stress levels corresponding to different axial strains gradually decrease. According to Fig 2(b), when the confining pressure is 100 kPa, as the axial strain increases, the deviatoric stress curves under different moisture content conditions show a trend of increasing first, then decreasing and gradually stabilizing, but the peak values of deviatoric stress corresponding to each working condition are significantly different. Among them, when the moisture content is 16%, the value of deviatoric stress is the largest, and the maximum shear strength can reach 150 kPa. According to Fig 2(c), when the confining pressure is 200 kPa, as the axial strain increases, the deviatoric stress under different moisture contents shows a gradually increasing trend. When the moisture content is 14%, the deviatoric stress is the maximum working condition, and the maximum deviatoric stress level is close to 300 kPa. As the moisture content gradually increases, the deviatoric stress level corresponding to axial strain gradually decreases. As shown in Fig 2(d), when the confining pressure is 300 kPa, the deviatoric stress under different moisture contents gradually increases. When the axial strain is greater than 7%, the deviatoric stress level gradually tends to be stable (the deviatoric stress level slightly reduces when the moisture content is 18%). When the moisture content is 14%, the deviatoric stress level reaches its maximum, and the maximum deviatoric stress is close to 750 kPa. As shown in Fig 2(a) and 2(b), the weak expansive soil has a peak when the strain is about 5%. According to Fig 2(c) and 2(d), for soil samples with the same moisture content, the larger the confining pressure, the larger the peak value of the stress difference.

### 4.2 Pore pressure changes

**Fig 3:**
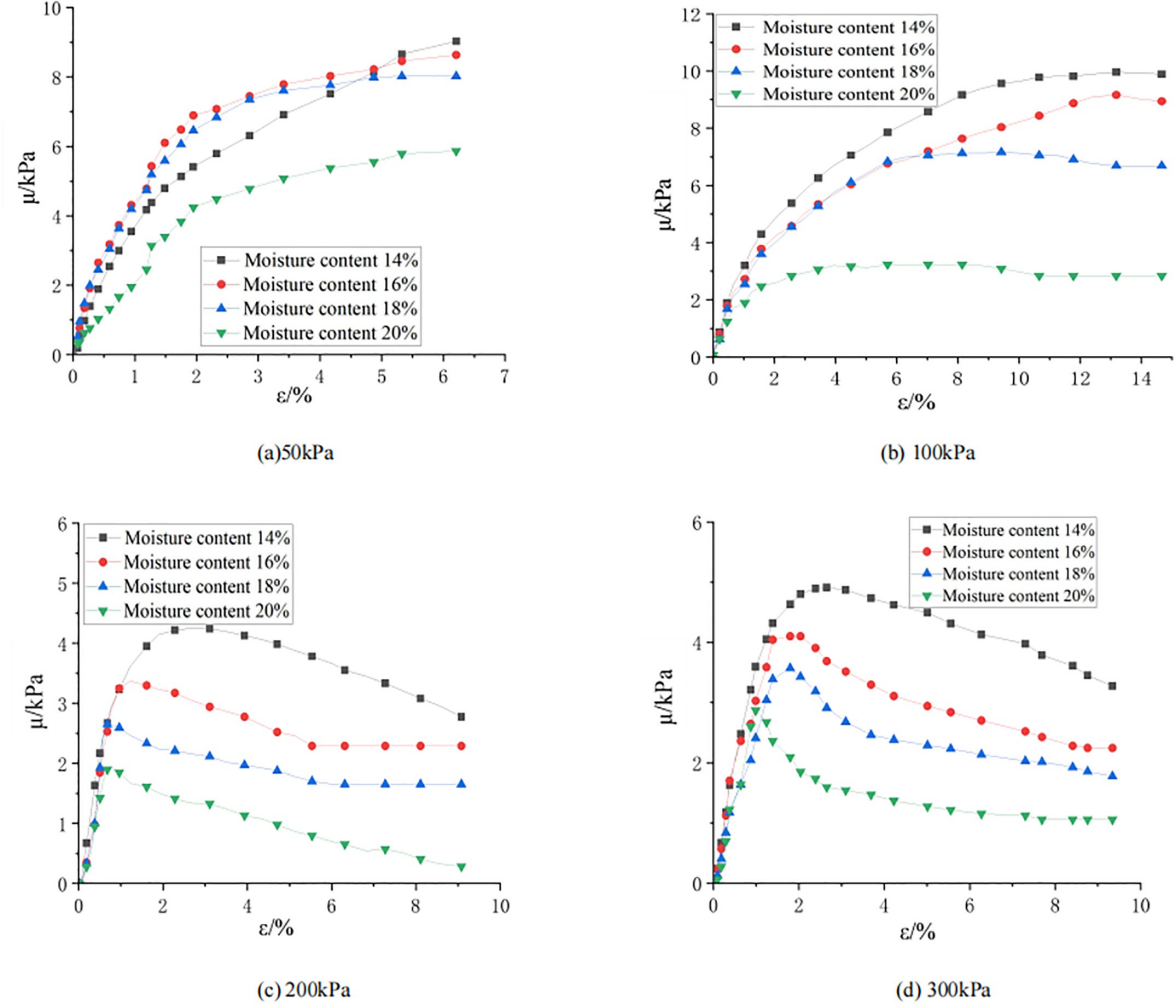
Pore pressure-strain diagram under different confining pressures.

The consolidated undrained shear test was used in this paper. Fig 3 shows the development laws of excess pore water pressure μ with axial strain ε under the same confining pressure and different moisture contents. As shown in Fig 3(a), when the confining pressure is 50 kPa, as the axial strain increases, the excess pore pressure under different moisture contents gradually increases and tends to be stable. When the moisture content is 16%, the excess pore pressure corresponding to each axial strain is relatively high, and the peak value of excess pore pressure is 9 kPa. As shown in Fig 3(b), when the confining pressure is 100 kPa, the excess pore pressure increases as the axial strain increases. When the moisture content is 14%, the excess pore pressure is the largest and the peak value of the excess pore pressure is 10 kPa. As the moisture content increases, the excess pore pressure corresponding to each axial strain decreases gradually. As shown in Fig 3(c) and 3(d), when the confining pressure is 200 kPa and 300 kPa, with the increase of the axial strain, the excess pore pressure under different moisture contents increases first and then decreases. Among them, when the moisture content is 14%, the pore pressure is the largest, and the peak value of excess pore pressure is 4.5 kPa. As the moisture content increases, the peak level of excess pore pressure decreases significantly. It can be seen that before the excess pore pressure reaches the peak, the internal particles of the sample are squeezed and deformed, and the compactness increases, thus increasing the pore pressure and shrinking the sample. After the excess pore pressure reaches a peak, the soil particles in the sample are staggered with each other, resulting in the volume expansion of the sample, and the shear expansion of the sample decreases the excess pore pressure. When the moisture content of the soil also increases, the moisture content inside the soil increases, thereby increasing the excess pore pressure in the soil.

### 4.3 Destructive forms

**Fig 4:**
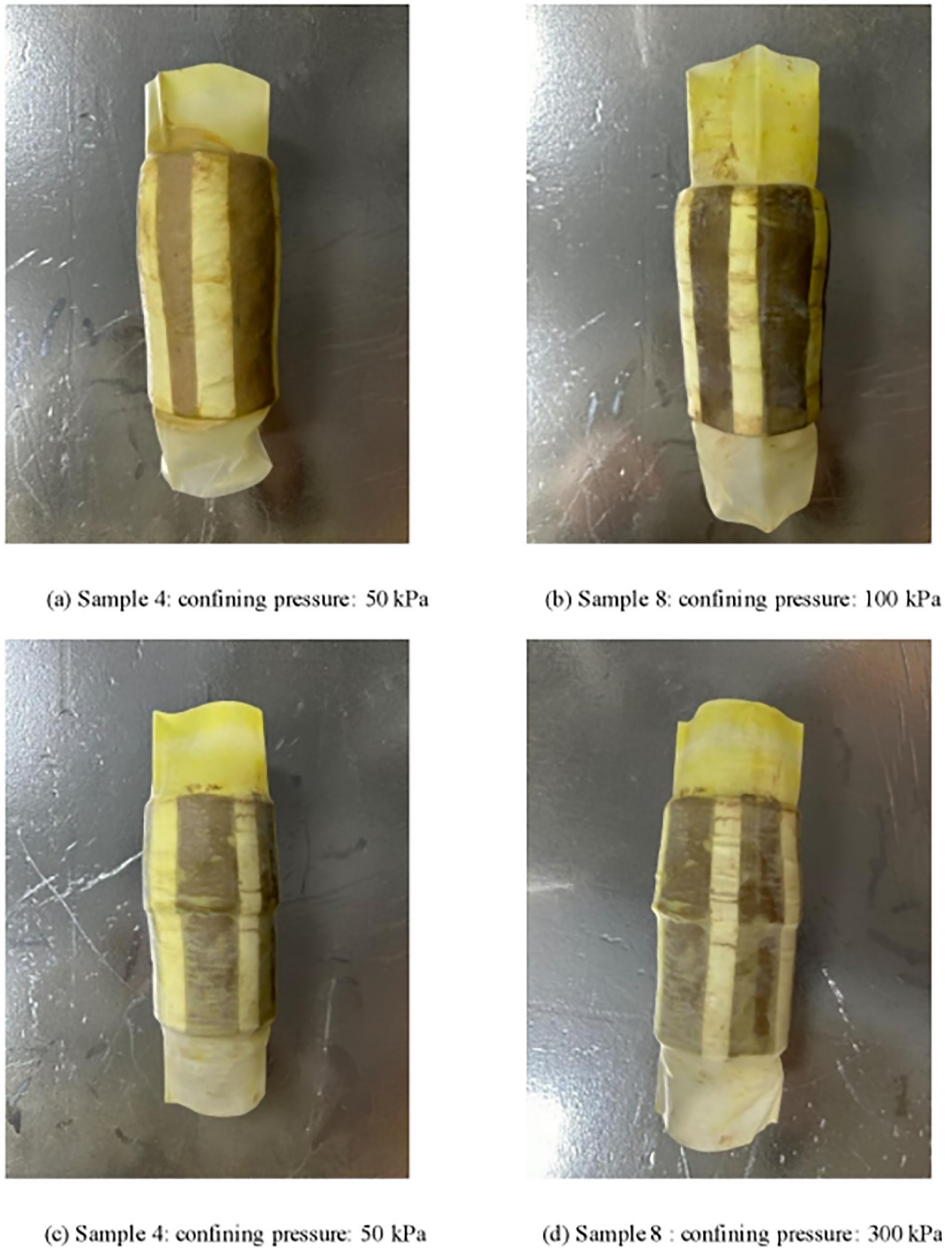
Soil samples during the testing process.

According to Section 4.2, when the moisture content is 20%, the deviatoric stress and excess pore pressure measured by triaxial specimens with different confining pressure levels are the smallest, indicating that the damage to the working condition test with a moisture content of 20% is the most significant and serious, which is consistent with the observation during the author’s test. Therefore, in this paper, the failure forms of weak expansive soil samples with a moisture content of 20% under different confining pressures were selected, as shown in Fig 4. Among them, Fig 4(a) shows the failure forms of soil samples under a confining pressure of 50 kPa. It is not difficult to see that the soil samples maintain the original form, only in the upper part of the drum shape deformation; Fig 4(b) shows the failure form of soil samples under the confining pressure of 100 kPa, and the drum deformation occurs in the upper and lower parts, which is the general failure form of weak expansive soil. Fig 4(c) and 4(d) show the failure forms of soil samples under a confining pressure of 200 kPa and 300 kPa, respectively, and there are obvious shear bands in the middle part. This is because the dilatancy of the sample expands the volume of some areas of the soil and reduces the compactness, so the strength of the soil in this part was reduced, and the shear failure occurred easily under large confining pressure.

### 4.4 Shear strength analysis

Under the action of triaxial external forces, most specimens undergo shear failure. Mohr believed that there are both shear stress and normal stress on the shear fracture surface, with the maximum shear stress and normal stress being a function relationship. Namely, *τ* = *τ*_0_ + *f*(*σ*)、*τ* is shear stress, *τ*_0_ is shear stress constant, *f* is friction coefficient, and *σ* is normal stress. According to the triaxial strength test, the stress value of the soil at failure can be obtained, thus obtaining the shear strength index. As the confining pressure increases, the cohesive force of expansive soil decreases, the internal friction angle increases, and the shear strength also gradually increases. The specific indicators are listed in the Table 4 below. We can observe that the greater the cohesion of soil, the smaller the internal friction angle, and the greater the shear strength.

**Table 4:** Shear strength indicators.

Confining pressure (kPa)	Cohesive force (kPa)	Internal friction angle (°)	Shearing strength (kPa)
50	42.93	23.87	65.06
100	38.30	26.25	87.61
200	33.95	26.87	135.28
300	12.21	30.06	185.83

## 5 Conclusion

This article investigates the stress-strain relationship, pore pressure, and other mechanical properties of weak expansive soil obtained from Nanyang weak expansive soil samples with different moisture contents of 14%, 16%, 18%, and 20%. Four sets of 16 consolidated undrained triaxial shear tests with confining pressures of 50100200300kPa were conducted to obtain the variation patterns of weak expansive soil under different loading conditions and moisture contents. The main conclusions are as follows:

(1) When under lower environmental pressure, the deviatoric stress-strain curve of weakly expansive soil shows an initial increase, followed by a decrease, and ultimately remains stable. Under high confining pressure conditions, as the axial strain increases, the deviatoric stress-strain curve of weakly expansive soil shows a non-linear increasing trend. (2) Under low confining pressure conditions, as the axial strain increases, the excess pore pressure curve of weakly expansive soil shows a non-linear increase and eventually tends to stabilize. When in a high pressure environment, as the axial stress increases, the overpressure curve shows a pattern of first increasing and then decreasing. And as the moisture content of the soil sample increases, the excess pore pressure level corresponding to the axial strain of low and high confining pressures gradually decreases. (3) Under low confining pressure, weakly expansive triaxial soil samples undergo shear shrinkage during the shear stage, and the soil structure is prone to failure. The phenomenon of deviatoric stress increasing first, then decreasing, and gradually stabilizing is observed; Under high confining pressure, weakly expansive triaxial soil samples experience shear dilation during the shear stage, making the soil less prone to failure. Due to the negative pore pressure generated during the shear dilation process, the excess pore pressure shows a trend of first increasing and then decreasing.
